# Physicochemical and histopathological parameters of broilers with dorsal cranial myopathy

**DOI:** 10.5713/ab.22.0109

**Published:** 2022-11-13

**Authors:** Ana Clara Longhi Pavanello, Fernanda Jéssica Mendonça, Thalita Evani Silva Oliveira, Guilherme Baú Torezan, Giovana Wingeter Di Santis, Adriana Lourenço Soares

**Affiliations:** 1 Department of Food Science and Technology, Universidade Estadual de Londrina, Londrina, PR 86057-970, Brazil; 2 Laboratory of Animal Pathology, Department of Veterinary Preventive Medicine, Universidade Estadual de Londrina, Londrina, PR 86057-970, Brazil; 3 Lar Cooperativa Agroindustrial, Rolândia, PR 86600-001, Brazil

**Keywords:** *Anterior latissimus dorsi* Muscle, Collagen, Fatty Acid Profile, Histology, Meat Quality

## Abstract

**Objective:**

This study aimed to investigate the effect of dorsal cranial myopathy (DCM) on chicken meat quality.

**Methods:**

Sixty-six Ross 308 AP broilers, 47 days old, of both sexes, weighing about 3.51 kg, were slaughtered according to standard industrial practices, and evaluated for meat color, pH, chemical composition, collagen content, fatty acid profile, and histopathological parameters. Comparisons between normal and DCM-affected meat were performed using Student's *t*-test at the 5% significance level.

**Results:**

Histological analysis of muscle tissues affected by DCM showed myofiber degeneration, proliferation of inflammatory cells, fibroplasia, and necrosis with fibrosis. DCM samples had lower protein content and higher moisture, ash, insoluble collagen, total collagen, and pH. DCM-affected meat was redder and more yellowish. There were no differences in lipid or soluble collagen contents between groups. DCM-affected meat had higher percentages of arachidonic acid (C20:4n−6) and eicosapentaenoic acid (C20:5n−3).

**Conclusion:**

This study revealed that DCM-affected meat exhibits considerable changes in quality parameters.

## INTRODUCTION

There has been a significant increase in the consumption of chicken meat in relation to other meat types, stimulating the poultry industry to improve its products and production processes. Nowadays, poultry production relies on the use of specially designed diets, intensive genetic selection, and well-controlled housing conditions, which allows obtaining birds with higher carcass yields in less time [1,2]. However, increased growth rate contributes to the development of several abnormalities in breast muscle tissues, such as white striping (WS), characterized by white striations parallel to muscle fibers [3]; wooden breast (WB), characterized by palpable hardness, pale areas, and caudal protuberance [4]; and spaghetti meat (SM), a structural alteration of intramuscular connective tissues that leads to the loosening of muscle fiber bundles [5]. These abnormal characteristics have a negative impact on technological and nutritional quality and are usually not well accepted by consumers because of appearance changes [6,7].

Another important muscle myopathy in birds is dorsal cranial myopathy (DCM), which affects the *anterior latissimus dorsi* (ALD) muscle and is characterized by yellowing and swelling of the skin. Affected ALD muscles exhibit subcutaneous edema, hemorrhagic areas, pallor, adhesion, and thickening. Histologically, this abnormality presents as polyphasic lesions with degenerative or necrotic fibers and fibrosis. DCM is responsible for a significant number of carcass condemnations in the poultry chain. Usually, the affected area, encompassing part of the wings and breast, has to be removed. While, in extreme cases, broilers presenting extensive lesions are condemned as a whole [8,9]. There are limited data available on this problem. DCM is responsible for at least 6% of the partial condemnations in Brazilian slaughterhouses. It is also a growing cause of carcass condemnation in slaughterhouses in the United States [10,11].

It is known that WS, WB, and SM myopathies may occur simultaneously [7]. DCM can also be associated with these abnormalities, given that their occurrence is related to high-performance strains and higher slaughter weights [7,8]. It must be noted, however, that the etiology of these muscle alterations is not yet fully established, reinforcing the need for further research to reduce losses in the poultry industry. There is also a lack of information on the relationship between physicochemical properties and histopathological parameters in affected ALD muscles. Therefore, considering the importance of understanding abnormalities in chicken meat, this study aimed to evaluate the effects of DCM on meat color, pH, chemical composition, collagen content, fatty acid profile, and histopathological parameters to minimize economic losses and improve the use of affected meat.

## MATERIALS AND METHODS

### Sampling procedures

Sampling was performed in a commercial processing plant located in the state of Paraná, Brazil. Broiler chickens from the same flock and a single strain (Ross 308 AP), 47 days old, of both sexes, weighing about 3.51 kg, were slaughtered according to standard industrial practices: hanging, electrical stunning, bleeding, scalding, defeathering, and evisceration. The Animal Ethics Committee at the State University of Londrina, Brazil, approved this research (protocol number 20962.2019.81).

**Figure 1:**
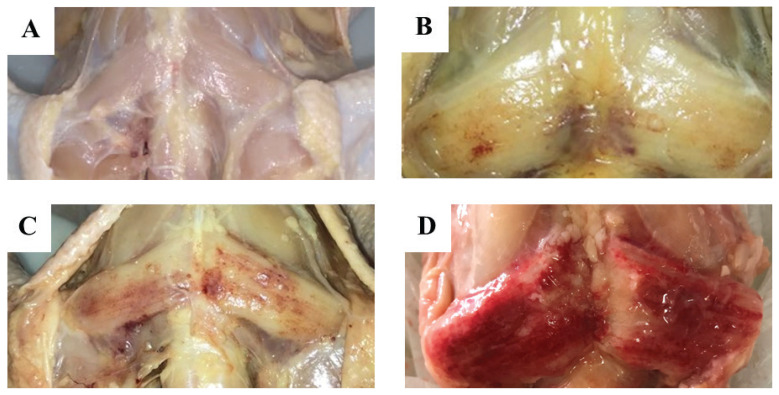
*Anterior latissimus dorsi* (ALD) muscles. (A) Normal ALD muscle. (B, C, D) ALD muscles affected by (B) moderate, (C) severe, and (D) extreme dorsal cranial myopathy.

ALD muscle samples were conveniently selected and collected from the federal inspection area. Samples were then classified into two groups through visual assessment by a trained specialist: i) DCM, samples exhibiting yellowish color between the wings, subcutaneous edema, and an odorless, gelatinous, citrine-yellow fluid at the affected site, and ii) normal, samples with no abnormalities. Figure 1 shows a representative image of DCM and normal muscles. DCM muscles were hardened, thick, and pale, with the surface partially or fully covered with scattered hemorrhages. After classification, normal samples (n = 28) and DCM samples (n = 28) were placed in plastic bags, labeled, maintained at 4°C for 24 h, and subjected to pH and color (L*, a*, b*) analyses. Samples collected for analysis of chemical composition, total and soluble collagen contents, and fatty acid profile were maintained at −18°C until use. Histological analysis was performed on samples collected immediately after slaughter.

### Histopathological evaluation

Longitudinal and transversal sections of normal and DCM-affected ALD muscles were prepared for histopathological analysis (5 samples per section per group, totaling 10 samples). The ends of muscle samples were pinned to a cork sheet to prevent muscle contractions. Muscle fragments were immersed in 10% buffered formalin solution, dehydrated with ethanol, diaphanized, and embedded in paraffin. Then, samples were routinely processed for histopathologic evaluation with hematoxylin and eosin stain. Masson's trichrome stain was used to identify connective tissue and fibrosis in affected muscle fibers. Lesions were identified, and microscopic morphological criteria were assessed based on the findings of Zimermann et al [8] and Sesterhenn et al [10]. Thus, hyaline myofiber degeneration was characterized by hypercontraction, hypereosinophilia, loss of polyhedral shape (rounding), sarcoplasmic homogenization, and size variations (cross-sections). Segmental necrosis was distinguished by cytoplasmic fragmentation of the myofibers acquiring a flocculent sarcoplasm (floccular necrosis) and macrophage and satellite cells migration into the myofiber. Regenerative findings included nuclear rowing on basophilic narrowed fibers. If edematous vascular granulation tissue and fibrin were found, fibroplasia was diagnosed, however, if proliferative collagenized connective tissue was present, fibrosis was identified. Inflammatory infiltrate was typified when present.

The microscopic findings were categorized (A, degenerations/necrosis; B, regeneration; C, fibroplasia; D, fibrosis; and E, inflammation) and semi-quantitatively scored from absent (0 points), mild (1 point), moderate (2 points) to severe (3 points). The final score was the sum of the individual scores in each category. The lowest score achievable was 0 and the highest was 15. Longitudinal and cross-sections from the same animal were analyzed together. In general, higher scores indicate more severe processes and lower scores indicate milder processes. The combination of different categories indicated polyphasic lesions.

### pH measurement

pH measurements were performed in duplicate at 24 h post-mortem using a pH meter with an insertion electrode (Testo 205, Lenzkirch, Germany).

### Color measurement

Color measurements were performed after removing the skin in two points (at different extremities of the ALD muscle) of samples at 24 h post-mortem [12] using a colorimeter (CR400; Konica Minolta, Japan) with CIE-D65 illuminant and CIE 10° standard observer. L*, a*, and b* parameters were measured in the CIELab color system.

### Chemical composition

Chemical composition of normal (n = 11) and affected (n = 11) ALD muscles were analyzed according to AOAC methods [13]. Moisture was determined after drying 5 g of sample in oven at 105°C to constant weight. Ash was determined by incineration of 5 g of samples in a muffle furnace at 550°C. Lipids were extracted from 1 g of sample in a Soxhlet with petroleum ether. Protein content was determined (0.2 g) by total nitrogen quantification by the Kjeldahl method, using a conversion factor of 6.25.

### Collagen content

#### Total collagen content

Total collagen content was determined of normal (n = 9) and DCM (n = 9) samples according to the method of Woessner Jr [14]. Initially, 0.5 g of sample was hydrolyzed with 15 mL of 6 mol/L HCl solution at 105°C. The hydrolysate was filtered, and the pH adjusted to between 6.0 and 7.0 with 50% (w/v) NaOH. The volume was completed to 250 mL with distilled water. Hydroxyproline concentration was determined after an oxidative reaction with chloramine T and perchloric acid, followed by complexation with *p*-dimethylaminobenzaldehyde. The absorbance was measured at 557 nm on a spectrophotometer. The standard curve was constructed with hydroxyproline (H1637; Sigma-Aldrich, Saint Louis, MO, USA), in the concentration range of 0.5 to 5 μg/mL. The total collagen was calculated from hydroxyproline content using a coefficient of 8.0 [15].

#### Soluble collagen content

Soluble collagen of normal (n = 9) and DCM (n = 9) samples was extracted according to the modified method of Penfield and Meyer [16]. A 2.0 g of sample was homogenized with 20 mL of deionized water for 1 min. Then, it was heated in a water bath at 80°C for 60 min. Samples were homogenized at 7,500×*g* using a Turratec TE-102 agitator (Tecnal TE-102, Piracicaba, Brazil) and centrifuged for 15 min at 4,000×*g*. The supernatant was filtered, and 30 mL of 6.0 mol/L HCl solution was added for hydrolysis. Quantification was performed according to the procedure described for total collagen determination.

### Fatty acid profile

Lipids were extracted according to the method of Bligh, E.G. and Dyer [17], with modifications. Briefly, 10 g of crushed sample of normal (n = 7) and affected (n = 7) was homogenized in 6 mL of methanol and 12 mL of chloroform for 5 min. Then, the mixture received the addition of 6 mL of chloroform and was homogenized for 2 min. Distilled water (6 mL) was added, and homogenization continued for 5 min. The homogenate was filtered and transferred to a separation funnel, to which 10 mL of saturated NaCl solution was added. After phase separation, the lower phase containing chloroform and fatty matter was collected, and the solvent was evaporated in a rotary evaporator (37°C±2°C) (801; Fisatom, Perdizes, Brazil). Transesterification of fatty acids was achieved according to method 5509 of the International Organization for Standardization [18], using 2 mol/L KOH in methanol and *n*-heptane.

Fatty acid methyl esters (FAMEs) were analyzed using a Shimadzu GC-17A gas chromatograph equipped with a flame-ionization detector and a capillary column (100 m× 0.25 mm) containing 0.25 μm particles in cyanopropyl polysiloxane (CP Sil 88). The column temperature program was as follows: 65°C for 15 min, from 65°C to 165°C at 10°C/min, held at 165°C for 2 min, from 165°C to 185°C at 4°C/min, held at 185°C for 8 min, from 185°C to 235°C at 4°C/min, and held at 235°C for 5 min. Detector and injector temperatures were set at 260°C, and the split ratio was 1/100. The gas flow rates were 1.2 mL/min for the carrier gas (H_2_), 30 mL/min for the auxiliary gas (N_2_), and 30 and 300 mL/min for flame gases (H_2_ and synthetic air, respectively). Fatty acids were identified by comparing relative retention times of peaks to those of FAME standards (Supelco, Bellafonte, PA, USA). Results are expressed as percentage of normalized area of the fatty acid peak.

### Statistical analysis

The results were submitted to the Shapiro–Wilk normality test (α = 0.05). To compare normal and DCM samples, the parametric Student's *t*-test (p<0.05) was applied, using the software Statistica for Windows version 7.0.

## RESULTS AND DISCUSSION

### Histopathological evaluation

**Figure 2:**
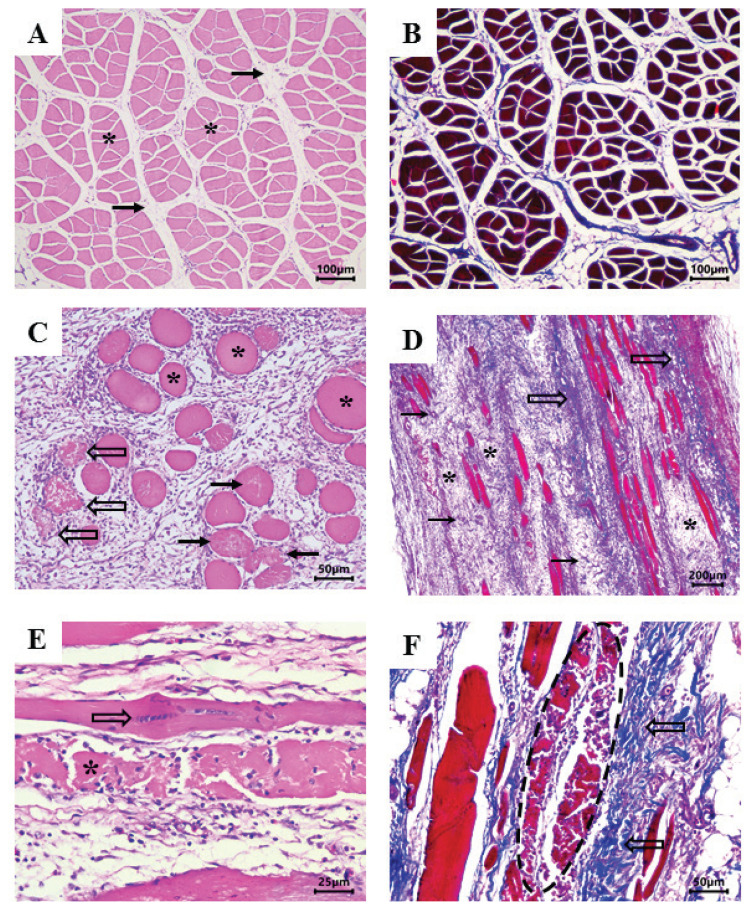
*Anterior latissimus dorsi* (ALD) muscle of broilers. (A, B) Normal skeletal muscle. (A) Transversal section showing bundles of myofibers with a polygonal pattern (asterisk) surrounded by a delicate perimysium with moderate fatty tissue infiltration (arrow). Hematoxylin and eosin (HE), 10×. (B) Same sample shown in panel A. Note the distinction between connective tissue (blue) and muscle cells (red). Masson’s trichrome, 10×. (C, D, E, F) Muscles affected by dorsal cranial myopathy. (C) Transversal section showing a prevalence of degenerated myofibers (asterisk), with variable size, rounded shape, and increased cytoplasmic eosinophilia. Some cells exhibit discrete cytoplasmic fragmentation (arrow), whereas others are markedly fragmented, to the point of losing their normal morphology, and are surrounded by an infiltrate of inflammatory cells (outline arrows). Note the spaced-apart myofibers with endomysial and perimysial proliferation of loose connective tissue. HE, 20×. (D) Longitudinal section showing loss of large amounts of myofibers and marked fibroplasia between the remaining muscle cells, characterized by proliferation of loose, swollen connective tissue (asterisk) and neoformed vessels (arrow), with some highly collagenized fibrotic focuses (stained in blue, outline arrows). Masson’s trichrome, 4×. (E) Longitudinal section showing myofibers in regeneration, with reduced diameter and several central nuclei (outline arrow), overlying the necrotic myocyte, markedly fragmented, and infiltrated by inflammatory cells. HE, 40×. (F) Longitudinal section showing necrotic myofibers, fragmented (dashed circle), adjacent to perimysial connective tissue fibrosis (outline arrows). Masson’s trichrome, 20×.

Important histopathological changes (Figure 2) were observed in ALD affected by DCM, which were associated with macroscopic lesions in tissues (Figure 1). Microscopically, ALD lesions exhibited degenerated myofibers surrounded by inflammatory cells (heterophils and macrophages mainly), with loss of polygonal pattern and increased cytoplasmic eosinophilia (Figure 2C). Sesterhenn et al [10] also observed the loss of polyhedral pattern and presence of hypercontraction, hypereosinophilia, sarcoplasmic homogenization, and inflammatory infiltration (predominantly mononuclear) in DCM samples. In assessing other myopathies (WS, WB, and SM), Soglia et al [19] and Baldi et al [5] observed nuclear internalization, loss of cross striations, variable cross-sectional areas (degenerating and regenerating fibers), lipidosis, proliferating connective tissue, and fibrosis.

Normal ALD muscle samples also presented microscopic lesions. Although, the scores were lower (from 1 to 4) than DCM-affected muscles (from 3 to 10), as previously observed by other authors [8,10]. These could represent a continuous pattern, early degenerative primary lesions could happen repeatedly inducing polyphasic late lesions, either by continuity of the primary causes or by the self-perpetuation of the lesion. Therefore, the lesions identified presented the following distribution: normal muscle 4 samples showed degenerations/necrosis (1 point each one), none with regeneration, 1 sample with fibroplasia (1 point), none samples with fibrosis, and 4 samples with inflammation (3 samples with 1 point and 1 sample with 2 points), while DCM muscle presented 5 samples with degenerations/necrosis (3 samples with 2 points and 2 samples with 3 points), 5 samples with regeneration (1 point each one), 3 samples with fibroplasia (2 samples with 1 point and 1 sample with 3 points), 3 samples with fibrosis (2 samples with 1 point and 1 sample with 2 points) and 5 samples with inflammation (3 samples with 2 points, 1 sample with 3 points and 1 sample with 1 point).

DCM samples were found to have the highest microscopic lesional scores, mainly due to degeneration/necrosis, causing loss of myofibers, inflammatory response, and fibroplasia or fibrosis between remaining muscle cells, characterized by proliferation of loose, edematous (immature) connective tissue or collagenized mature connective tissue, respectively (Figure 2D, 2F). Fibrosis has been associated with the macroscopic hardness of WB fillets [4]. Geronimo et al [20] confirmed that the raw WB fillets were tougher than unaffected samples by the shear force instrumental measure. Therefore, the fibrosis process could also explain the macroscopic hardness of affected ALD muscles. In some cases, it was possible to identify regenerating myofibers with reduced diameter and several central nuclei (Figure 2E). By contrast, tissues from normal samples did not show inflammatory cells or degenerative/necrotic changes, only moderate infiltration of fatty tissues (Figure 2A, B).

These findings indicate that DCM is a polyphasic process. According to Zimermann et al [8], polyphasic lesions in chickens might be associated with the occurrence of an initial lesion followed by consecutive damage related to increased growth rate and body size.

### Color and pH

DCM-affected ALD muscles showed higher (p<0.05) pH than normal samples (Table 1). This result is in agreement with that observed in other myopathies (WS, WB, and SM) affecting chicken breast meat [5,21,22]. Previous studies correlated high growth rate and muscle yield in chickens with high final pH, attributed to alterations in glycolytic potential [23]. Meat affected by abnormalities has lower glycolytic potential, and, consequently, post-mortem reduction of pH is slower, resulting in higher final pH values than in normal meat [22,24].

Regarding meat color (Table 1), L* (lightness) was lower (p<0.05) in DCM samples, which is probably related to the macroscopic lesions found in affected tissues (Figure 1). The parameters a* (redness/greenness) and b* (yellowness/blueness) were higher (p<0.05) in DCM than in normal samples, indicating a redder and more yellowish surface. Yellowish color is correlated with the presence of abnormalities, such as WB, as a consequence of the fibrotic response occurring in affected muscles [20,24]. These color changes are associated with superficial hemorrhagic lesions and presence of a citrus yellow gelatinous fluid, characteristic of DCM [8], confirming that DCM affects muscle appearance (Figure 1).

### Chemical composition

The presence of DCM influenced (p<0.05) meat chemical composition (Table 2). Compared with normal samples, DCM samples showed high moisture and ash contents and low protein content (p<0.05). Baldi et al [25], Soglia et al [26], and Geronimo et al [20] reported higher moisture and lower protein contents in meat affected by WS and WB.

The increase in moisture is probably related to liquid accumulation (moderate to severe edema) as a result of inflammatory processes [4,26]. The reduction in protein content in abnormal meat has been associated with myodegeneration and reorganization of muscle tissue, resulting in replacement of muscle fibers with fat and connective tissues [3,20,26]. Here, we did not observe differences (p≥0.05) in lipid content between samples, in contrast to the results of Soglia et al [26] and Geronimo et al [20] for WS and WB fillets.

In a previous study on turkey breast affected by a severe degree of WS, Carvalho et al [27] found higher ash contents in affected samples (normal = 1.67 g/100 g, WS = 1.81 g/100 g). Soglia et al [26], on the other hand, found reduced ash content in chicken breast affected by WB and WB/WS (normal = 1.37 g/100 g, WB = 1.26 g/100 g, WB/WS = 1.21 g/100 g).

### Collagen content

DCM increased (p<0.05) the concentration of total and insoluble collagen in muscles (Table 3) but did not alter soluble collagen content (p≥0.05). Collagen function is related to structure; the protein contributes to tissue shape and mechanical properties [28]. No literature data were found on the collagen content of DCM meat. However, the results are consistent with reports of other abnormalities in chicken meat. Mudalal et al [21] and Geronimo et al [20], in studying WS and WB, reported that total collagen content was higher in affected meat than in normal samples. Hasegawa et al [29] observed that muscles affected by WB have twice the total collagen content of normal meat.

Such changes are related to myodegeneration, muscle tissue regeneration, and deposition of interstitial connective tissue or fibrosis (Figure 2), which contribute to excessive formation of cross-links, making collagen more insoluble and, consequently, increasing meat rigidity [26,30]. Sanden et al [31] observed that WB samples were rich in connective tissue, in which thin and thick collagen fibers occurred in a disarranged mixture, whereas normal fillets contained mature thick collagen fiber bundles packed in organized structures. The authors concluded that the high hardness of raw WB meat is attributed to extensive deposition of connective tissue. Thus, in the current study, the high (p<0.05) insoluble and total collagen contents of DCM samples may explain the hard consistency of affected ALD muscles.

Collagen/protein ratio was higher (p<0.05) in DCM samples, suggesting reduced nutritional value.

### Fatty acid profile

Fatty acid profiles are reported in Table 4. Overall, DCM did not influence (p≥0.05) saturated, monounsaturated, or polyunsaturated fatty acid contents. However, C20:4n−6 (arachidonic acid) and C20:5n−3 (eicosapentaenoic acid) contents were higher (p<0.05) in DCM-affected ALD muscles. These findings may be related to inflammatory responses, as previously discussed (Figure 2C), which are usually associated with a higher proportion of arachidonic acid [32].

Soglia et al [33] found that WB fillets had a higher level of Ca^2+^. An increase in Ca^2+^ may promote degenerative processes through activation of phospholipase A_2_, a lipolytic enzyme that affects the phospholipid membrane, releasing unsaturated fatty acids, mainly arachidonic acid [34,35]. The specific mineral content of DCM muscles is unknown. Here, we observed that ash content was higher in DCM samples (Table 2). The increase in ash content could be related to an increase in Ca^2+^ content.

The action of eicosapentaenoic acid (n−3) is opposite to the inflammatory effects of arachidonic acid (n−6) [32]. These polyunsaturated fatty acids are produced and accumulated on the basis of tissue needs [36]. Higher amounts of eicosapentaenoic acid in DCM samples might therefore be associated with an attempt to regulate inflammatory processes and maintain homeostasis. Further studies are needed to elucidate the factors and mechanisms underlying the increase in fatty acid concentrations in poultry meat affected by DCM.

## CONCLUSION

DCM causes significant changes in the physicochemical, structural, and histopathological characteristics of meat. DCM-affected muscles showed fragmented myofibers with excessive fibroplasia, necrosis, and fibrosis, resulting in meat with a notably redder and more yellowish color. DCM samples had higher moisture, ash, and collagen contents and reduced protein concentration. As a consequence, muscle consistency was considerably affected. Higher percentages of arachidonic and eicosapentaenoic acids were observed in DCM samples, indicating an inflammatory response. The magnitude of the effects of DCM demonstrated that this abnormality is an important quality issue in the poultry industry.

## Figures and Tables

**Table 1:** Color parameters (L*, a*, and b*) and pH values of normal chicken meat and meat affected by dorsal cranial myopathy (DCM)

Parameters	NORM (n = 25)	DCM (n = 25)
pH	6.25^b^±0.09	6.31^a^±0.08
L*^1)^	63.91^a^±1.77	58.78^b^±2.83
a*^1)^	6.64^b^±0.75	12.68^a^±3.11
b*^1)^	8.26^b^±1.78	14.16^a^±2.46

Values are presented as mean±standard deviation.

1) L*, lightness; a*, redness/greenness; b*, yellowness/blueness.

a,b Means in the same row followed by different lowercase letters are significantly different (Student's *t*-test, p≤0.05).

**Table 2:** Chemical composition of normal chicken meat and meat affected by dorsal cranial myopathy (DCM).

Parameters (g/100 g)	NORM (n = 11)	DCM (n = 11)
Moisture	75.73^b^±1.14	77.12^a^±1.60
Ashes	0.64^b^±0.05	0.73^a^±0.03
Proteins	17.84^a^±0.67	16.01^b^±1.10
Lipids	9.93^a^±2.06	8.36^a^±1.52

Values are presented as mean±standard deviation.

a,b Means in the same row followed by different lowercase letters are significantly different (Student’s *t*-test, p<0.05).

**Table 3:** Total, soluble, and insoluble collagen contents of normal chicken meat and meat affected by dorsal cranial myopathy (DCM)

Parameters	NORM (n = 9)	DCM (n = 9)
Total collagen (g/100 g)	1.35^b^±0.15	1.55^a^±0.20
Soluble collagen (g/100 g)	0.12^a^±0.02	0.12^a^±0.04
Insoluble collagen (g/100 g)	1.24^b^±0.14	1.43^a^±0.18
Collagen/protein ratio	7.58^b^	9.67^a^

Values are presented as mean±standard deviation.

a,b Means in the same row followed by different lowercase letters are significantly different (Student's *t*-test, p≤0.05).

**Table 4:** Fatty acid profile of normal chicken meat and meat affected by dorsal cranial myopathy (DCM)

Fatty acid (%)	NORM (n = 7)	DCM (n = 7)
C14:0 (myristic)	0.53^a^±0.00	0.50^a^±0.00
C16:0 (palmitic)	18.68^a^±0.04	21.17^a^±0.01
C16:1 (palmitoleic)	4.09^a^±0.01	3.67^a^±0.01
C18:0 (stearic)	5.25^a^±0.02	6.53^a^±0.01
C18:1n−9 (oleic)	39.45^a^±0.03	36.98^a^±0.01
C18:2*n*−6 (linoleic)	28.65^a^±0.02	27.79^a^±0.02
C20:1*n*−9 (gadoleic)	0.16^a^±0.00	0.11^a^±0.00
C18:3*n*−3 (α-linolenic)	2.39^a^±0.00	2.16^a^±0.00
C20:2*n*−6 (eicosadienoic)	0.16^a^±0.00	0.18^a^±0.00
C20:3*n*−6 (dihomo-γ-linolenic)	0.16^a^±0.00	0.15^a^±0.00
C20:4*n*−6 (arachidonic)	0.38^b^±0.00	0.55^a^±0.00
C20:5*n*−3 (EPA)	0.12^b^±0.00	0.18^a^±0.00
∑SFA	24.46^a^±0.06	28.21^a^±0.01
∑MUFA	43.69^a^±0.04	41.00^a^±0.01
∑PUFA	31.85^a^±0.02	31.00^a^±0.02
∑PUFA/SFA	1.40^a^±0.49	1.10^a^±0.11
*n*−6/*n*−3	11.76^a^±0.50	12.28^a^±0.63

Values are presented as mean±standard deviation.

EPA, eicosapentaenoic acid, SFA, saturated fatty acids, MUFA, monounsaturated fatty acids, PUFA, polyunsaturated fatty acids.

a,b Means in the same row followed by different lowercase letters are significantly different (Student's *t*-test, p≤ 0.05).
